# Abundance and Genetic Diversity of Microbial Polygalacturonase and Pectate Lyase in the Sheep Rumen Ecosystem

**DOI:** 10.1371/journal.pone.0040940

**Published:** 2012-07-17

**Authors:** Peng Yuan, Kun Meng, Yaru Wang, Huiying Luo, Huoqing Huang, Pengjun Shi, Yingguo Bai, Peilong Yang, Bin Yao

**Affiliations:** Key Laboratory for Feed Biotechnology of the Ministry of Agriculture, Feed Research Institute, Chinese Academy of Agricultural Sciences, Beijing, People’s Republic of China; Belgian Nuclear Research Centre SCK/CEN, Belgium

## Abstract

**Background:**

Efficient degradation of pectin in the rumen is necessary for plant-based feed utilization. The objective of this study was to characterize the diversity, abundance, and functions of pectinases from microorganisms in the sheep rumen.

**Methodology/Principal Findings:**

A total of 103 unique fragments of polygalacturonase (PF00295) and pectate lyase (PF00544 and PF09492) genes were retrieved from microbial DNA in the rumen of a Small Tail Han sheep, and 66% of the sequences of these fragments had low identities (<65%) with known sequences. Phylogenetic tree building separated the PF00295, PF00544, and PF09492 sequences into five, three, and three clades, respectively. Cellulolytic and noncellulolytic *Butyrivibrio*, *Prevotella*, and *Fibrobacter* species were the major sources of the pectinases. The two most abundant pectate lyase genes were cloned, and their protein products, expressed in *Escherichia coli*, were characterized. Both enzymes probably act extracellularly as their nucleotide sequences contained signal sequences, and they had optimal activities at the ruminal physiological temperature and complementary pH-dependent activity profiles.

**Conclusion/Significance:**

This study reveals the specificity, diversity, and abundance of pectinases in the rumen ecosystem and provides two additional ruminal pectinases for potential industrial use under physiological conditions.

## Introduction

The natural diet of ruminants is mainly plant material. The fibrous cell walls of plants contain three major types of polysaccharides: cellulose, hemicellulose and pectin. Cellulose is an assembly of long unbranched fibrils each made of ∼30 to 36 intermolecularly hydrogen-bonded chains of β-1,4-glucose. Hemicellulose is a branched, non-charged polysaccharide that forms hydrogen bonds with cellulose. Pectin contains, by definition, linear α-1,4-linked d-galacturonic acid residues plus other sugars [1]. Ruminants digest vegetation by initially softening it within the first stomach (rumen), then regurgitating the semi-digested mass and chewing it again [2]. This process by which vegetation is broken down and digestion is stimulated involves action by ruminal microbes [3], [4].

**Figure 1:**
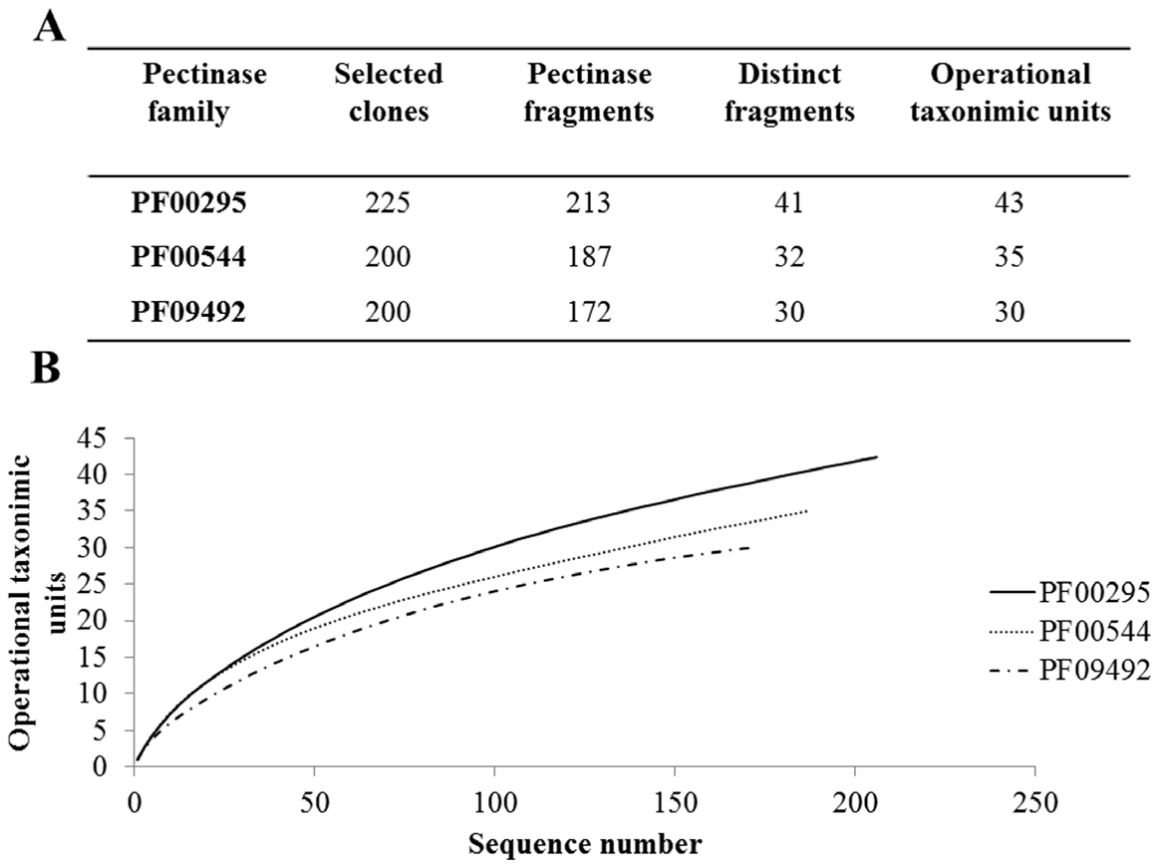
Characterization of the pectinase gene-fragments from microbial DNA in the rumen of a Small Tail Han sheep. **A** Summary of the selected clones of PF00295, PF00544 and PF09492 libraries. **B** Rarefaction curves created by DOTUR. The gene types (OTUs) were defined by a 6% distance separation.

The ruminal microbial ecosystem contains obligate anaerobic bacteria, fungi, protozoa and methanogenic archaea [5]. These microbes secret many different enzymes that degrade plant cell walls and therefore represent an important resource for hydrolytic carbohydrate-specific enzymes [6]. The genetic diversity and composition of microbes in the rumen of sheep, cows, and camels have been explored by sequencing or pyrosequencing 16 S/18 S rDNA [7]–[9], denaturing gradient gel electrophoresis of 16 S rDNA [10], restriction fragment length polymorphism characterization of 16 S rRNA [11] and suppressive subtractive hybridization of total genomic DNA [12]. To date, however, only a few studies have explored the functional diversity of polysaccharide-degrading enzymes from ruminal microbes. Ferrer *et al*. identified 22 glycoside hydrolases in a metagenome library constructed from bovine ruminal microflora [13], Huang *et al*. retrieved 101 cysteine phytase gene fragments from the ruminal genomic DNA of Boer goats and Holstein cows [14], and Wang *et al*. cloned 87 xylanase microbial gene fragments from the goat rumen [15]. These studies revealed the diversity and abundance of ruminal hydrolytic enzymes.

Pectin is an important determinant of plant cell-wall porosity and thickness as it glues cells together into an adhesive layer (the middle lamella) [16]. It is the major carbohydrate present in legumes and in some non-forage plants and it constitutes 10 to 20% of total carbohydrate in grass and alfalfa [17]. Pectin degradation accelerates cellulose and hemicellulose degradation and maintains the physiological pH of the rumen [18]. Appreciable digestion of pectin (75 to 90%) has been observed in sheep [19]. Mixed cultures of ruminal bacteria can ferment isolated pectin [20], [21]. Pectinase is the general term for an enzyme that degrades pectin, and these enzymes are widely distributed in microorganisms and plants. According to their catalytic mechanisms and tertiary structures, pectinases can be grouped into three classes: pectin esterases, protopectinases, and depolymerases. Pectin esterases catalyze the deesterification of the methyl ester linkages of the pectin galacturonan backbone and release acidic pectin and methanol. Protopectinases liberate water-soluble and highly polymerized pectin from protopectin. Depending on their mode of action, depolymerases act as polygalacturonases and cleave the α-1,4 glycosidic bonds between two non-esterified galacturonate units or as pectate lyases that cleave glycosidic bonds via β-elimination [22], [23]. Polygalacturonases and pectate lyases are more commonly found in the rumen than are pectin esterases and protopectinases [24], [25]. Some important ruminal cellulose- and hemicellulose-digesting bacteria, e.g., *Ruminococcus albus* (EGC02601.1), *Fibrobacter succinogenes* (ACX76067.1), *Butyrivibrio proteoclasticus* (ADL35238.1) and *Prevotella ruminicola* (EFU31294.1), secrete polygalacturonases and pectate lyases [26].

**Table 1:** Conserved amino acid motifs in the five pectinase families and the sequences of the degenerate primers designed accordingly to retrieve pectinase gene fragments from ruminal microbial genomes.

Pectinasefamily	Conserved motif(5′ → 3′)	Primer (5′ → 3′)^a^	
PF00295	GDDC(H)I(V)AI(V)K	GGCGAYGAYTGYRTNGCAITNAA	
	GL(I)RIKS	CGGWCTTGATYCTNAKNCC	
PF00544	GTHV(I)WI(V)DH	GGTACGCACRTNTGGRTNGAYCA	
	HV(A)Y(V)NNYYE	CTCGTAGTARTTRTTRTAIRCRTG	
PF03211	W(F)EDV(I)G(C)ED	GTGGGAGGACRTNKGNGARGA	
	GKLY(L)RSCG	GCCACAGGAGCGNWANAGYTTICC	
PF06917	GLLYWGGH	GGNCTNCTNTAYTGGGGNGGNCA	
	DGYYGKKG	CCCTTCTTGCCRTARTANCCRTC	
PF09492	AQYPNGGW(F)	GCTCAGTACCCNAAYGGNGGNT	
	WA(G)QQH(Y)DE	CTCGTCGTRYTGYTGNSCCCA	

a Y  =  C/T, K  =  T/G, R  =  A/G, N  =  A/T/G/C.

The goal of this study, using a PCR-based approach, was to characterize the abundance and diversity of the major polygalacturonases and pectate lyases that were present in the rumen of the Small Tail Han sheep mainly fed on corn stalk, wheat bran, and grasses. The results obtained expand our knowledge of ruminal pectinases and provide two newly identified pectinases that may find use in industrial applications performed under physiological conditions.

## Materials and Methods

### Ethics Statement

All animal studies were followed the regulation for the review committee of laboratory animal welfare and ethics and protocol for the review on laboratory animal welfare and ethics, Beijing Administration Office of Laboratory Animal. The animal experimentation was approved by the Committee of Laboratory Animal Welfare and Ethics, Beijing Administration Office of Laboratory Animal with the approval No. SYXK2008-0007.

**Figure 2:**
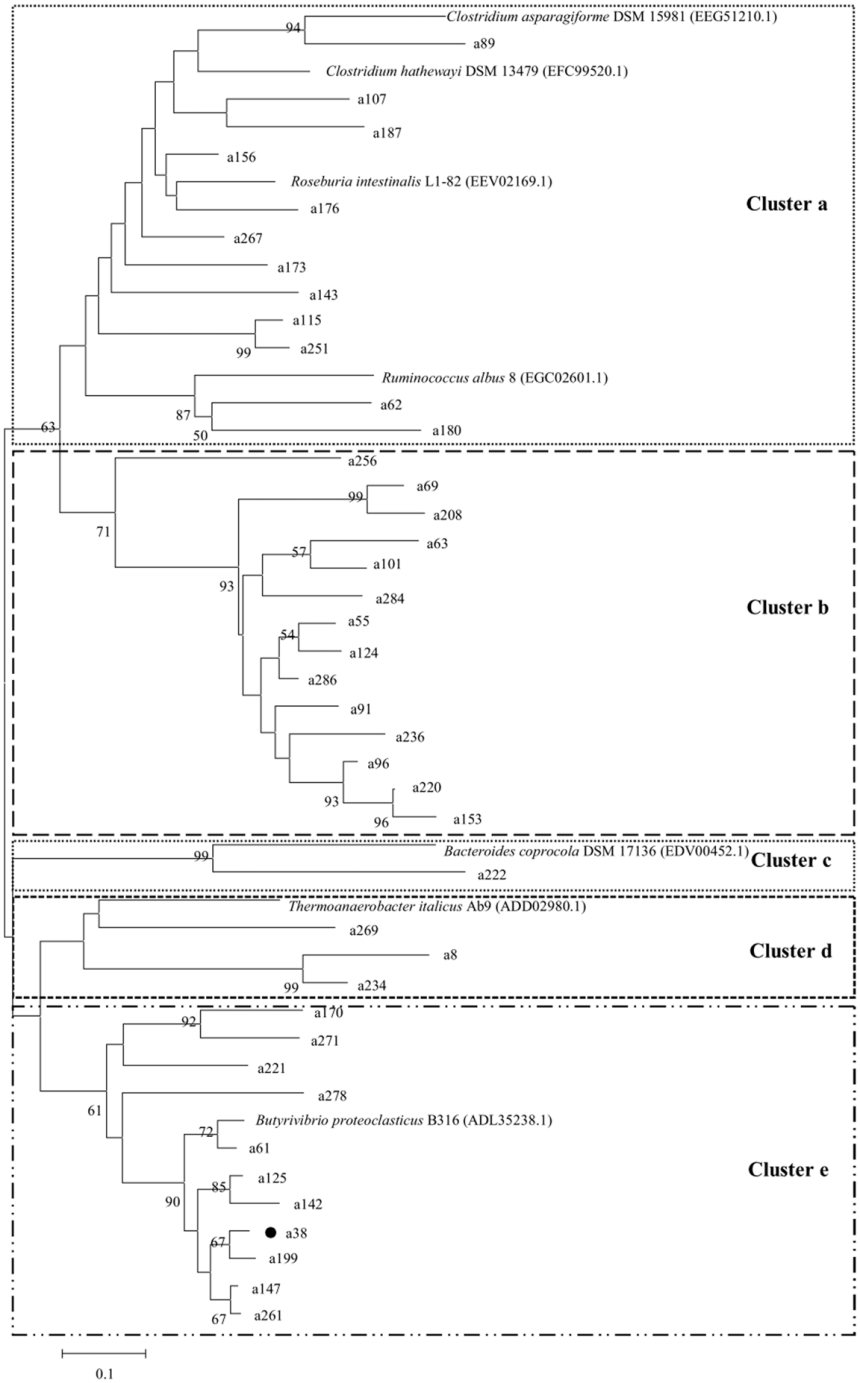
Phylogenetic tree build using the partial amino acid sequences derived from the microbial PF00295 polygalacturonase gene fragments from the rumen of the Small Tail Han sheep referenced to polygalacturonase sequences retrieved from GenBank. The tree was constructed using the neighbor-joining method (MEGA 4.0). The lengths of the branches indicate the relative divergence between pairs of amino acid sequences. The numbers at the nodes are bootstrap values based on 1000 bootstrap replications. Only bootstrap values >50 are displayed. The scale bar represents 0.1 amino acid substitution per position. The reference source strains and their GenBank accession numbers are identified. The highest abundance fragment is marked by a dot (•).

### Rumen Content Sampling and Total DNA Extraction

Three two-year-old Small Tail Han sheep (Sheep 2, Sheep 3 and Sheep 7) fed on corn stalk, wheat bran and grasses were used to collect ruminal fluid via gastric tubes at specific time points over a period of 24 h after their morning feeding. Ruminal fluids were centrifuged at 5000×*g*, 4°C for 30 min. The supernatant fractions were subjected to polygalacturonase and pectate lyase activity assays as described below. Because polygalacturonase and pectate lyase activities were found to be maximum around six hours after feeding (Figure S1), this time was taken for subsequent analyses.

The rumen content at 6 h post-feeding of one arbitrarily chosen Small Tail Han sheep was immediately filtered through four layers of cheesecloth and centrifugation (17,000×*g*, 4°C, 30 min) [14], [15]. The pellet was stored at –70°C until used. Samples of the pellet were ground in liquid nitrogen to a fine powder with a mortar. Total genomic DNA was extracted following a protocol specific for high molecular weight, environmental DNA [27] and purified using the reagents of a TaKaRa Agarose Gel DNA Purification kit (Tokyo, Japan).

**Figure 3:**
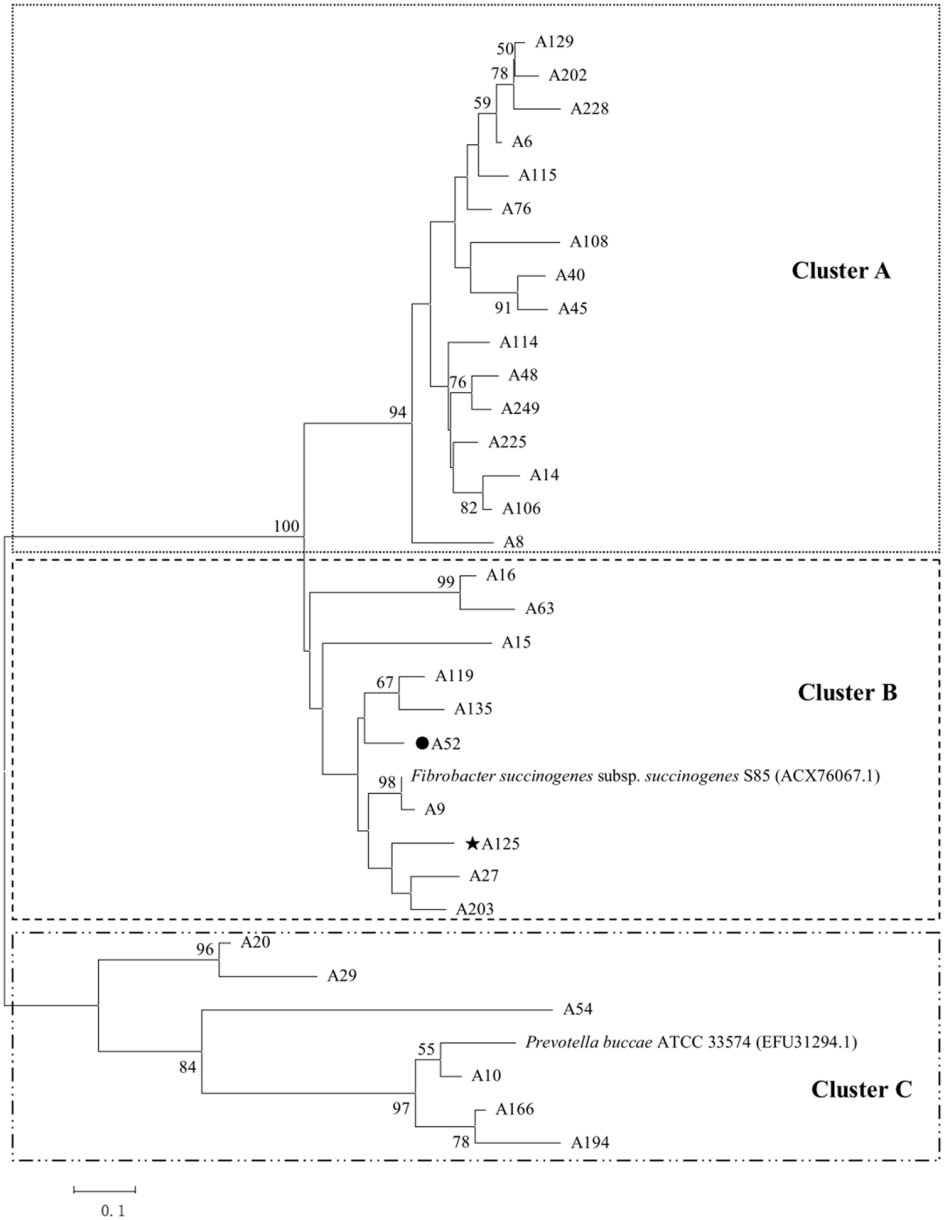
Phylogenetic tree build using the partial amino acid sequences derived from the microbial PF00544 pectate lyase gene fragments from the rumen of the Small Tail Han sheep referenced to pectate lyase sequences retrieved from GenBank. The tree was built as described in Figure 2. The most abundant fragment is marked by a dot (•). The gene fragment used to retrieve *A125* is marked with a star (★).

**Figure 4:**
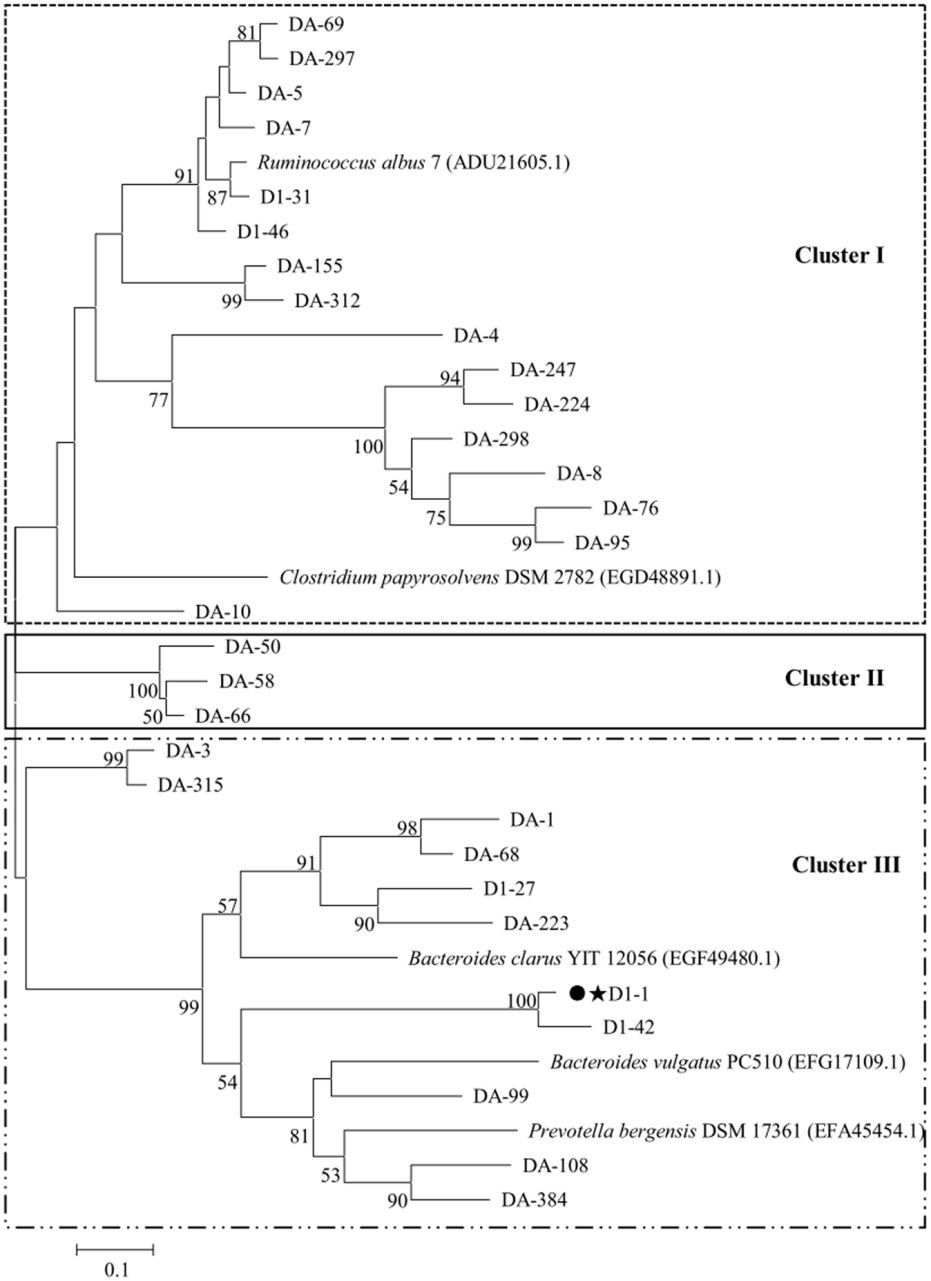
Phylogenetic tree build using the partial amino acid sequences derived from the microbial PF09492 pectate lyase gene fragments from the rumen of the Small Tail Han sheep referenced to pectate lyase sequences retrieved from GenBank. The tree was built as described in Figure 2. The most abundant fragment that was used to retrieve *D1-1* is marked by a dot (•) and a star (★).

### Primer Design, PCR Amplification, and Primer Verification

The Pfam database is a large collection of protein sequences grouped by family, which includes multiple sequence alignments and Hidden Markov Models (HMM) logos for each family [28]. The database of Pfam release 25.0 contains one polygalacturonase family (PF00295) and four families of pectate lyases (PF00544, PF03211, PF06917, PF09492) for a total of 3358 microbial pectinase sequences (Up to Dec 2, 2011). We identified the conserved regions of each family sequences and designed five degenerate primer sets according to COnsensus-DEgenerate Hybrid Oligonucleotide Primer design principles [29]. To verify that the design of the primers was appropriate, eight representative strains that are evolutionarily separated were selected, i.e., the Gram-positive bacteria *Alicyclobacillus* sp. A4, *Streptomyces* sp. ACCC41168, and *Bacillus* sp. CGMCC 1.2016, the Gram-negative bacteria *Xanthomonas* sp. ACCC10048 and *Klebsiella* sp. Y1, and the fungi *Penicillium* sp. CGMCC 1669, *Bispora antennata* CBS 126.38 and *Aspergillus flavus* sp. J4 (Table S1). The genomic DNAs of these strains were extracted and used as templates. PCR amplification used the following program: 95°C for 5 min; 94°C for 30 s followed by 52°C for polygalacturonase or 55°C for pectate lyase for 30 s, then 72°C for 30 s (this cycle was repeated 10 times with the temperature of the second step decreased 0.5°C each cycle); 40 cycles of 94°C for 30 s, 47°C for polygalacturonase or 50°C for pectate lyase for 30 s, and 72°C for 30 s; and a final extension step at 72°C for 10 min. The PCR products were visualized by ethidium bromide staining after electrophoresis through 1.5% (w/v) agarose gels. Those products of the correct size were purified with the reagents of a TaKaRa DNA Purification kit.

**Figure 5:**
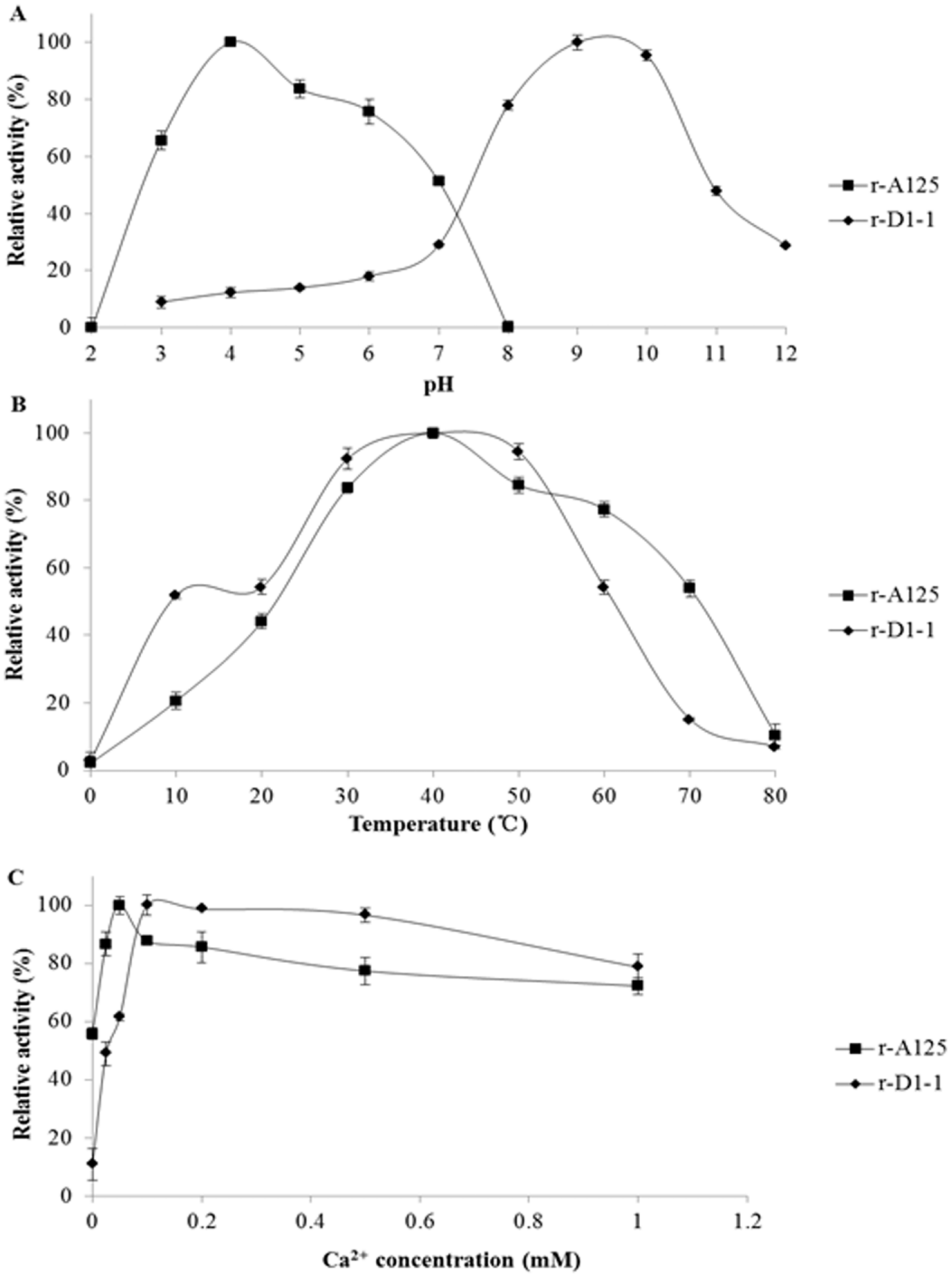
A125 and D1-1 temperature, pH and Ca^2+^ dependencies. **A** pH-dependent activity profiles for A125 and D1-1 at 40°C. **B** Temperature-dependency profiles for A125 and D1-1 activities. Activities were measured using 100 mM citric acid–Na_2_HPO_4_ (pH 4.0) and 100 mM Tris–HCl (pH 9.0) for A125 and D1-1, respectively. **C** Ca^2+^-dependency profiles for A125 and D1-1. Each value is the mean ± standard deviation (*n* = 3).

### Construction of the Clone Libraries

Pectinase gene fragments were amplified by touchdown PCR as described above using the purified microbial DNA from the sheep rumen as template and 5 primer sets specific for each gene of PF00295, PF00544, PF03211, PF06917, and PF09492 families. Only objective gene products of PF00295, PF00544, and PF09492 families were retrieved from the sheep rumen DNA. Purified PCR products with the correct sizes were each ligated into a pGEM-T Easy vector (Promega) and then individually transformed into *Escherichia coli* JM109 cells (TaKaRa) by electroporation. Cells were grown on agar plates that contained Luria Bertani medium, 100 µg ml^–1^ ampicillin, 80 µg ml^–1^ 5-bromo-4-chloro-3-indolyl-β-d-galactopyranoside, and 0.5 mM isopropyl-thio-β-D-galactopyranoside at 37°C overnight. For preliminary characterization of the PCR products, 50 positive transformants (white clones) from each pectinase family were randomly chosen, their inserts were PCR amplified using the M13F and M13R primers, and then sequenced. Their sequence similarities were assessed by BLASTx (http://www.ncbi.nlm.nih.gov/BLAST/). The fragments that appeared most often in the 50 clones from each pectinase library were identified by multiple sequence alignment using ClustalX.

To comprehensively evaluate the diversity of pectinase genes in sheep rumen and prevent abundant fragments to overshadow low-abundance genes as indicated by Bohannana and Hughes [30], the most abundant gene fragments of each library were removed. Primer a-cut R (Table S2) specific for the most abundant fragment, a38, of PF00295 family was designed and combined with primer M13F to remove a38 from the pool of PF00295 PCR products. PCR amplification conditions were 26 cycles at 94°C for 30 s, 54°C for 30 s, and 72°C for 30 s; followed by one extension step at 72°C for 10 min. The most abundant fragment of PF09492 family was removed by a restriction digest with *Ava*I (CYCGRG; TaKaRa) at 37°C for 4 h. After the PCR reaction or the restriction digest, the products were electrophoresed in 1.5% agarose gels for recovery. The pool of PF00544 PCR products was not dominated by any fragment.

The recovered PF00295 and PF09492 PCR products and the PF00544 PCR products of the expected sizes were each ligated into pGEM-T Easy to generate three clone libraries as described above. More than 200 clones from each pectinase family (Figure 1A) were then sequenced at Biomed (Beijing, China).

### Sequence Analysis

The nucleotide sequences of pectinase gene fragments were translated into amino acid sequences by EMBOSS Transeq (http://www.ebi.ac.uk/emboss/transeq) after determining the correct reading frames. Redundant amino acid sequences were removed by CD-hit using a 95% sequence-identity cutoff. The protein sequence similarities were assessed by BLASTp (http://www.ncbi.nlm.nih.gov/BLAST/). Multiple sequence alignment of the sequences in each pectinase family was performed by ClustalX. Phylogenetic trees were constructed by MEGA 4.0 [31] using the neighbor-joining method [32]. Tree topology confidence levels were assessed using the bootstrap values from 1000 replicates. Fourteen representative pectinase sequences from the NCBI database were selected and used as references for tree construction. Rarefaction curves were calculated for the pectinases in each family using distance-based operational taxonomic units (OTUs) and richness determination (DOTUR) software [33]. Distance matrices for the protein fragments were calculated using the default parameters of PROTDIST in PHYLIP (http://evolution.genetics.washington.edu/phylip.html). Sequences were then assigned to OTUs on the basis of UPGMA (average linkage clustering) implemented in DOTUR with default parameters of precision (0.01) and bootstrap (1000 replicates); rarefaction results for 6% protdist were calculated. The nucleotide and amino acid sequences of full-length genes were assessed by BLASTx and BLASTp, respectively. SignalP (http://www.cbs.dtu.dk/services/SignalP/) was used to predict potential signal peptides of proteins.

### Cloning of Full-length Pectate Lyase Genes

To obtain the full-length mature pectinase genes, *A125* and *D1-1*, the 5′ and 3′ fragments flanking the core regions were amplified by TAIL−PCR [14], [34] with twelve nested insertion-specific primers (Table S2) and the reagents of a Genome Walking kit (TaKaRa). PCR products of the expected sizes were electrophoresed through 1.2% agarose gels, isolated, individually cloned into pGEM-T Easy vectors, sequenced, and assembled with known fragment sequences to form complete open reading frames. The nucleotide and amino acid sequences were assessed by BLASTx and BLASTp, respectively. The two open reading frames were PCR amplified using the primer sets A125-F/R and D1-F/R (Table S2) and sheep ruminal template DNA, then individually cloned into pGEM-T Easy and sequenced.

### Expression and Characterization of the Pectate Lyases A125 and D1-1

After digestion with *Nco*I and *Not*I, *A125* and *D1-1* were individually cloned behind a T7 promoter/lac operator in vector pET-22b(+). The recombinant plasmids, pET-*A125* and pET-*D1-1*, were transformed into *E. coli* BL21 (DE3) competent cells. Positive transformants were identified by restriction digestion and DNA sequencing, and then one colony for each gene was grown in Luria Bertani medium containing 100 µg ml^−1^ ampicillin at 37°C to an A_600_ of ∼0.6. Protein expression was induced by addition of isopropyl-thio-β-D-galactopyranoside (final concentration, 500 µM) for 48 h at 20°C.

Pectate lyase activity at 40°C was determined by measuring the absorbance change at 235 nm for 30 min [35], [36] for solutions that contained enzyme and 0.2% (w/v) polygalacturonic acid (substrate) in a buffer containing 100 mM citric acid–Na_2_HPO_4_ (pH 4.0) and 50 µM CaCl_2_ or in a buffer containing 100 mM Tris–HCl (pH 9.0) and 100 µM CaCl_2_. One activity unit (U) was defined as the amount of enzyme that produced 1 µmol of unsaturated galacturonide per minute. The galacturonide concentration was calculated using a molecular extinction coefficient at 235 nm of 4600/M/cm [35]. All activity assays were performed in triplicate.

Polygalacturonic acid was used as the substrate to characterize the dependence of activity on temperature and pH. pH-dependent activity profiles were determined at 40°C in buffers of 100 mM citric acid–Na_2_HPO_4_ (pH 2.0–7.0), 100 mM Tris–HCl (pH 8.0–10.0) or 100 mM glycine–NaOH (pH 11.0–12.0). To determine the optimal temperatures, the activities were measured between 0°C and 80°C on the optimal pH. The dependence of A125 and D1-1 activities on Ca^2+^ concentration was determined between 0 and 1 mM CaCl_2_.

### Nucleotide Sequence Accession Numbers

The nucleotide sequences of the pectinase gene fragments were deposited into the GenBank database under the accession numbers JQ282281–JQ282383. Accession numbers JQ265996 and JQ265997 were assigned to the full-length pectate lyase genes *A125* and *D1-1*, respectively.

## Results

### Design and Verification of Degenerate Primers Specific for Pectinase Genes

According to the Pfam release 25.0 and the HMM logo profiles and primer design principles described in Method section, five degenerate primer sets were designed specific for genes of PF00544, PF03211, PF06917, and PF09492 families (Table 1). PCR products of the expected size (180 base pairs (bp) for PF00295, 270 bp for PF00544, 180 bp for PF03211, 850 bp for PF06917, and 270 bp for PF09492) were obtained when these primers and template genomic DNA from representative pectinase-producing strains with significant evolutionary separation were used (Table S1). All products were identified as fragments of pectinase genes by sequencing and BLAST characterization. When the ruminal microbial DNA from the Small Tail Han sheep was used as the template with the primer sets for PF00295, PF00544, and PF09492, the expected fragments were amplified. Conversely, amplified DNA was not recovered when the PF03211 and PF06917 primer sets were used. Further analysis of the PF03211 and PF06917 pectate lyses indicated that they are all non-ruminal metazoal, fungal, or bacterial in nature.

### PCR Amplification and Preliminary Product Characterization

To construct clone libraries, pectinase gene fragments were PCR amplified using the ruminal microbial DNA from the sheep as the template and the PF00295, PF00544, and PF09492 primer sets. Fifty positive transformants (white colonies) from each library were randomly chosen for confirmation by PCR with primers M13F and M13R. Of the 50 positive clones from each library, 48 PF00295, 47 PF00544, and 47 PF09492 clones that contained an insert of the correct size (209 bp for PF00295, 268 bp for PF00544 and 266 bp for PF09492, respectively) were sequenced. No introns were identified. According to the BLASTx examination, the 142 cloned sequences had 45–99% amino acid identity with sequences of known pectinases. The most abundant pectinase gene fragments were a38 (21/45) for PF00295, A52 (9/44) for PF00544 and D1-1 (27/43) for PF09492.

### Removal of the Most Abundant Gene Fragments and Construction of Clone Libraries

Using procedures of PCR amplification with primers M13F and a-cut R or restriction digest with endonuclease *Ava*I, the most abundant fragments a38 of PF 00295 family and D1-1 of PF09492 family were removed from the pools of PCR products. Due to its relatively low abundance, fragment A52 of PF00544 family was retained for further analysis. All targeted gene fragments were recovered from the gels and each ligated into a pGEM-T-Easy vector for library construction. Over 5000 clones were prepared for each library, and 625 positive clones from the three libraries were randomly selected for confirmation by PCR as described above (Figure 1A). BLAST analysis indicated that the sequences of 572 fragments in the 625 clones (91.5%) contained a correct reading frame and were 45 to 99% identical with pectinase sequences found in GenBank. The sequences of these cloned fragments were credited to be identified or unidentified pectinases.

### Amino Acid Sequence Alignment and Abundance Analysis

According to their correct reading frames, the partial pectinase gene sequences, 213 from PF00295, 187 from PF00544 and 172 from PF09492, were translated into amino acid sequences by EMBOSS Transeq, respectively. The deduced amino acid sequences in each family were aligned by ClustalX. The highly conserved His residues in the polygalacturonases from PF00295 (corresponding to His-322 of the *Ruminococcus albus* 8 polygalacturonase sequence) and the pectate lyases from PF00544 (corresponding to His-443 of the *F. succinogenes* subsp. *succinogenes* S85 pectate lyase), and the highly conserved Asp residue of the pectate lyases from PF09492 (corresponding to Asp-166 of *Bacteroides vulgatus* PC519), were found in the respective sequences (Figure S2, S3, S4). Those completely conserved residues were assumed to be related with catalytic and substrate binding [37], [38].

After removing redundant sequences with CD-hit [39], the remaining 103 sequences (41 for PF00295, 32 for PF00544, and 30 for PF09492; Figure 1A) represented a set of evolutionarily divergent sequences as they had <95% identities with those of the GenBank pectinases (Tables S3, S4, S5). Abundance levels determined using distance-based OTUs and DOTUR software showed that a38, A52, and D1-1 represented the most commonly found gene fragments in each family (Tables S3, S4, S5). A rarefaction curve for each of the three pectinase families was created using a 6% distance (Figure 1B). The 572 pectinase gene fragments represented 108 OTUs. Of them, 82.9% of the PF00295 OTUs, 66.7% of the PF00544 OTUs, and 80.0% of the PF09492 OTUs were <80% identical with those of known pectinases (Table S6). Moreover, 65.6% of the OTUs had <65% identity with known sequences. Therefore, it appears that a large number of the pectinase gene sequences discovered in this study originate from an abundance of unknown (and yet to be cultured) ruminal organisms.

### Diversity Analysis

Three phylogenetic trees were constructed as described by Dubey *et al*. [23] using the aforementioned 103 distinct sequences and 14 reference pectinase sequences from the NCBI protein database that had sequence identities <95% with each other and with the 103 sequences. From what follows, most of the reference sequences are from ruminal bacteria. There was substantial diversity in the pectinase sequences, i.e., five clades (a, b, c, d, e), three clades (A, B, C), and three clades (I, II, III), which are distinct evolutionary clades for PF00295, PF00544, and PF09492, respectively (Figures 2, 3, 4). Except for 14 of the PF00295 polygalacturonase sequences that had no close homologs, the other PF00295 sequences were related to polygalacturonases from Bacteroidetes (29.3%; *Butyrivibrio* and *Bacteroides*) and Firmicutes (29.3%; *Clostridium*, *Ruminococcus*, and *Roseburia*). The main PF00544 pectate lyase producer was Fibrobacteres, as 31.3% of the PF00544 pectate lyase sequences were associated with this phylum. Approximately 18.8% of the PF00544 sequences were related to those from Bacteroidetes. High bootstrap values (1000 replicates) separated the PF09492 pectate lyase sequences into three clades, 53.3% were related to the pectate lyases from Firmicutes (*Clostridium* and *Ruminococcus*) and 36.7% from Bacteroidetes (*Bacteroides*). Considering the great abundance of a38 (46.7% of the PF00295 fragments) and D1-1 (62.8% of the PF09402 fragments), Bacteroidetes was the main producer of PF00295 and PF09402 pectinases indeed. A52 and A125 were the most abundant fragments found for PF00544 (27.8%) and were mostly produced by Fibrobacteres.

According to the phylogenetic analysis, the most abundant fragment a38 in the PF00295 library was closely related to a polygalacturonase from *B. proteoclasticus* B316 (ADL35238.1), which is a Gram-positive, polysaccharide-degrading anaerobic bacterium that was originally isolated from bovine rumen and is widely distributed in ruminants fed on diverse diets [40]. A52 and A125 were the predominant fragments in the PF00544 library. Their closest homolog is the important fibrolytic ruminal bacterium *F. succinogenes* subsp. *succinogenes* S85 (ACX76067.1) [41]. *F. succinogenes* appears to be the major ruminal cellulolytic species, as it accounts for as much as 7% of the bovine ruminal microbial mass [42]. D1-1 was the predominant sequence in the PF09492 library and shared high identity with the pectate lyase sequence from *B. vulgatus* PC510 (EFG17109.1). *Bacteroides* and *Prevotella* are the main determinant bacteria in rumen environment, which have been reported to account for as many as 60% of the ruminal bacterial isolates from silage-fed cows [43] and involve in plant cell-wall polysaccharide degradation [44]–[46].

### Gene Cloning, Expression, and Characterization of Pectate Lyases

Two full-length newly discovered pectate lyase genes, *A125* and *D1-1*, were cloned from the microbial genomic DNA in rumen of the Small Tail Han sheep by degenerate PCR and TAIL–PCR [14], [47]. *A125* contained 1698 bp that encoded 565 amino acids. A putative signal peptide sequence (residues 1–28) was predicted by SignalP with a reliability score of 0.869. The deduced A125 sequence had the greatest identity (87%) with that of the pectate lyase from *F. succinogenes* subsp. *succinogenes* S85. The 1122-bp *D1-1* encoded a mature protein of 355 residues plus a predicted 18-residue signal peptide (reliability score of 0.916). The deduced protein sequence was 44% identical with that of the pectate lyase from *B. vulgatus* ATCC 8482. Because both pectate lyases had signal peptides, they should be secreted directly into the rumen interior.

The proteins encoded by *A125* and *D1-1* in their mature forms were each expressed in *E. coli* BL21 (DE3) cells. After induction with isopropyl-thio-β-D-galactopyranoside at 20°C for 48 h, substantial pectate lyase activities were detected in the lysates of cells that harbored the plasmid pET-*A125* (0.2 U ml^–1^) or pET-*D1-1* (1.1 U ml^–1^). No pectinase activity was detected in cultures of uninduced transformants or transformants that harbored an empty pET-22b(+).

The pH optima for the pectate lyase activities of recombinant A125 and D1-1 were 4.0 and 9.0, respectively (Figure 5A). A125 exhibited 50–80% of its optimum activity between pH 5.0–7.0, which is the Han sheep ruminal pH range. Both pectate lyases had maximum activity at 40°C (Figure 5B), which is approximately the Han sheep ruminal temperature (39°C). Considering the great abundance and excellent activity-associated properties of A125, it might be the main determinant in the pectate lyase activity in Han sheep rumen. The addition of Ca^2+^ increased the activities of A125 and D1-1, with the optimal Ca^2+^ concentrations being 50 µM for A125 and 100 µM for D1-1 (Figure 5C).

## Discussion

Pectin is a major component of plant cell walls. Biodegradation of pectin is needed for ruminants to digest their food; this includes cellulose and hemicellulose degradation, and ruminal pH homeostasis [18]. Pectinases constitute a diverse group of enzymes and are widespread in nature [48]. To our knowledge, the work reported herein is the first to describe pectinase abundance and genetic diversity in the rumen of a sheep at the metagenomic level.

Of the five primer sets specific for the polygalacturonase and pectate-lyase families, only fragments from three families (PF00295, PF00544, PF09492) were PCR amplified when the genomic DNA from the sheep rumen served as the template. Our database and literature searches indicated that the other two pectate lyases families (PF03211 and PF06917) were from plant-pathogenic anaerobic bacteria (*Xanthomonas* and *Erwinia*) that cause soft-rot disease or were metazoan (such as *Meloidogyne* and *Globodera*), fungi (*Fusarium* and *Aspergillus*), or aerobic bacteria (*Streptomycineae*, *Bacillus*, *Pseudomonas*, and *Yersinia*) in origin, and these organisms have not been detected in the rumen. Therefore, the rumen may represent an unmined resource for non-pathogenic anaerobic bacterial pectinases.

Pectinase genes have been isolated from a number of anaerobic ruminal bacteria [13], [26], [49]; however, the genetic diversity and abundance of ruminal pectinases have not been reported prior to this study in which 103 distinct pectinase DNA fragments (41 from PF00295, 32 from PF00544, and 30 from PF09492) were cloned using three sets of degenerate primers and sheep ruminal DNA. These sequences represent uncharacterized pectinases, were phylogenetically diverse and varied in abundance. Of them, ∼7% of their OTU sequences were <50% identical with known sequences (including the most abundant fragment sequence D1-1 from the PF09492 library). The phylogenetic trees illustrated that the PF00544 and PF09492 fragment sequences were closely related to those of *Butyrivibrio* and *Bacteroides* and *Prevotella* pectate lyases, and the PF00295 fragment sequences were highly similar to those of *Fibrobacter* polygalacturonases. *Butyrivibrio*, *Bacteroides* and *Prevotella*, and *Fibrobacter* are well-known ruminal cellulolytic and non-cellulolytic bacteria [50]. Bacterial symbiosis and competition occur in the rumen, thus different types of pectinases from various bacteria allow pectin to be efficiently degraded in the rumen environment [51].

It is somewhat surprising that we did not retrieve pectinase gene fragments from ruminal anaerobic fungi and protozoa. Although ruminal fungi possess enzymes that can hydrolyze most plant cell-wall polysaccharides [52], there are fewer ruminal fungal species than bacterial species. The reasons why eukaryotic pectinases were not found might include the following reasons. (i) The pectinase genes from ruminal fungi and protozoa have introns in the DNA sequences of the conserved motifs that may have interfered with PCR amplification using the designed primers. (ii) The amount of eukaryotic template DNA was small, which has been overshadowed by bacterial DNA [30]. (iii) Because eukaryotic, ruminal pectinase sequences have yet to be submitted to the NCBI database, the database may not have provided the needed information for the BLASTx search [52]. In the future we intend to focus on characterizing eukaryotic, ruminal pectinases.

Until now, only a few pectinase genes (ADB80098, ACA61143 and ACZ98651) have been found by direct cloning of uncultured, ruminal microorganism genomes or by screening a ruminal metagenomic library (CAJ19131) [13], [26]. However, the gene products were not expressed or biochemically characterized. For our study, we cloned, expressed and biochemically characterized two sheep ruminal pectate lyases. Both enzymes were optimally active at 40°C, the ruminal temperature, and had complementary pH-dependent activity profiles that were wider than that found in the rumen (pH 5.5–7.0). The pH optima of A125 and D1-1 were 4.0 and 9.0, respectively, thereby covering a broad pH range (pH 3.0, >60% relative activity, to pH 11.0, >40% relative activity). Acidic cellulases that are most active between pH 4.0 and 5.0 have been identified in the rumen of buffalo, and these cellulases can degrade cellulose fibers at low pH [48]. Our results and those of Duan and colleagues [48] therefore suggest that the rumen of an individual animal harbors microbes that secrete various polysaccharide-specific enzymes so as to maximize the degradation of plant fibers.

In conclusion, we developed an efficient culture-independent and sequence-based molecular biological method to explore the diversity and abundance of ruminal pectinases in Small Tail Han sheep. As we were able to isolate a large number of pectinase gene fragments from Han sheep rumen, we postulate that pectinases play an important role in plant biomass degradation in these sheep. Three pectinase families were identified; each family contained high levels of fragments that were related to those from the anaerobic bacteria that dominate the Small Tail Han sheep ruminal ecosystem. By cloning the genes for two pectate lyases for which high levels of gene fragments had been found, and then heterologously expressing the gene products, we determined their activities in relation to the Small Tail Han sheep ruminal environment. As one of these is an acidic pectinase and the other is an alkaline pectinase, they may be used in combination or separately at physiological temperature for the preparation of juices, textiles, biofuels, and animal-feed products and for other agricultural applications.

## Supporting Information

Figure S1 **Pectinase activities of rumen fluids over 24 after morning feeding.** **A** Polygalacturonase activity assayed at 39°C and pH 6.5 by DNS method. **B** Pectate lyase activity assayed at 39°C and pH 6.5 by HCl method [45].
(TIF)

Figure S2 **Amino acid sequence alignment by ClustalX of pectinase fragments from clone library of PF00295 with the known polygalacturonase from** ***Ruminococcus albus*** **8 (EGC02601).** Identical and similar residues are shaded in black and gray, respectively. The highly conserved residue, His, is indicated with “*”.
(TIF)

Figure S3 **Amino acid sequence alignment by ClustalX of pectinase fragments from clone library of PF00544 with the known pectate lyase from** ***Fibrobacter succinogenes*** **subsp.** ***succinogenes*** **S85 (ACX76067).** Identical and similar residues are shaded in black and gray, respectively. The highly conserved residue, His, is indicated with“*”.
(TIF)

Figure S4 **Amino acid sequence alignment by ClustalX of pectinase fragments from clone library of PF09492 with the known pectate lyase from** ***Bacteroides vulgatus*** **PC510 (EFG17109).** Identical and similar residues are shaded in black and gray, respectively. The highly conserved residue, Asp, is indicated with “*”.
(TIF)

Table S1 PCR-mediated retrieval of known microbial DNA pectinase sequences to test the capacity of the designed primers to retrieve pectinase sequences from the sheep rumen ecosystem.
(DOC)

Table S2 Primers used in this study.
(DOC)

Table S3 Unique PF00295 polygalacturonase gene fragments retrieved from the microbial ecosystem of a Small Tail Han sheep rumen and their closest sequentially related relatives according to amino acid sequence identity.
(DOC)

Table S4 Unique PF00544 pectate lyase gene fragments retrieved from the microbial ecosystem of a Small Tail Han sheep rumen and their closest sequentially related relatives according to amino acid sequence identity.
(DOC)

Table S5 Unique PF09492 pectate lyase gene fragments retrieved from the microbial ecosystem in a Small Tail Han sheep rumen and their closest sequentially related relatives according to amino acid sequence identity.
(DOC)

Table S6 Percentages of total PF00295, PF00544, and PF09492 fragments (operational taxonomic units) that have amino acid sequence identities within a given range in comparison with GenBank pectinase sequences.
(DOC)
