# Supplementary figures and images for: Abundance and Genetic Diversity of Microbial Polygalacturonase and Pectate Lyase in the Sheep Rumen Ecosystem
Source: PLoS One. 2012 Jul 17;7(7):e40940. doi: 10.1371/journal.pone.0040940 (PMC3398870; doi:10.1371/journal.pone.0040940)

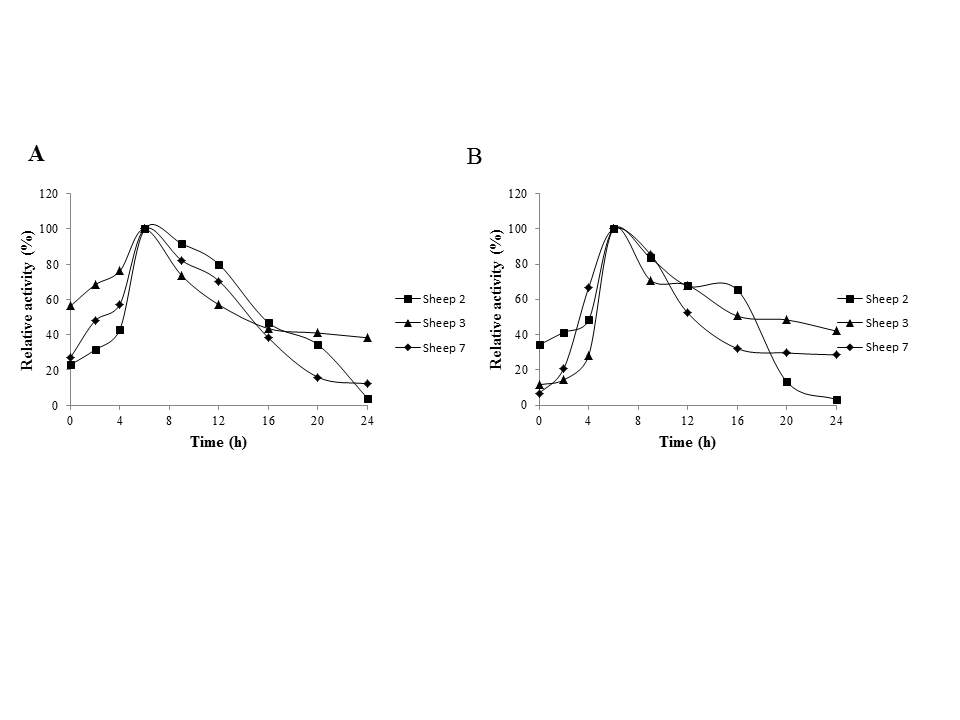

Supplement: Figure S1 — Pectinase activities of rumen fluids over 24 after morning feeding. A Polygalacturonase activity assayed at 39°C and pH 6.5 by DNS method. B Pectate lyase activity assayed at 39°C and pH 6.5 by HCl method [45]. (TIF) [file pone.0040940.s001.tif]

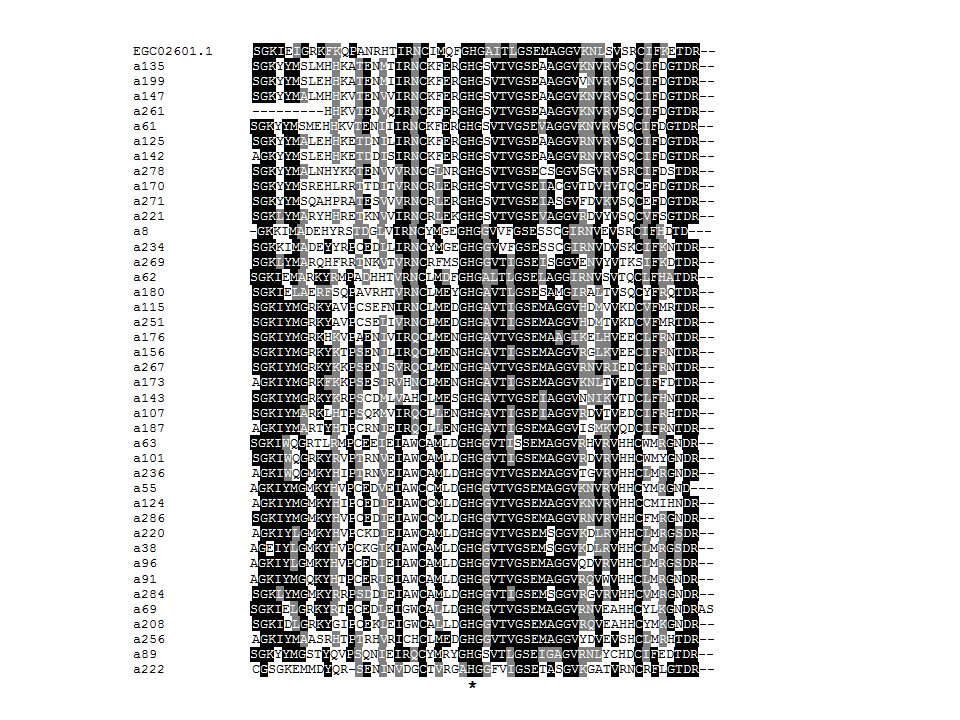

Supplement: Figure S2 — Amino acid sequence alignment by ClustalX of pectinase fragments from clone library of PF00295 with the known polygalacturonase from Ruminococcus albus 8 (EGC02601). Identical and similar residues are shaded in black and gray, respectively. The highly conserved residue, His, is indicated with “*”. (TIF) [file pone.0040940.s002.tif]

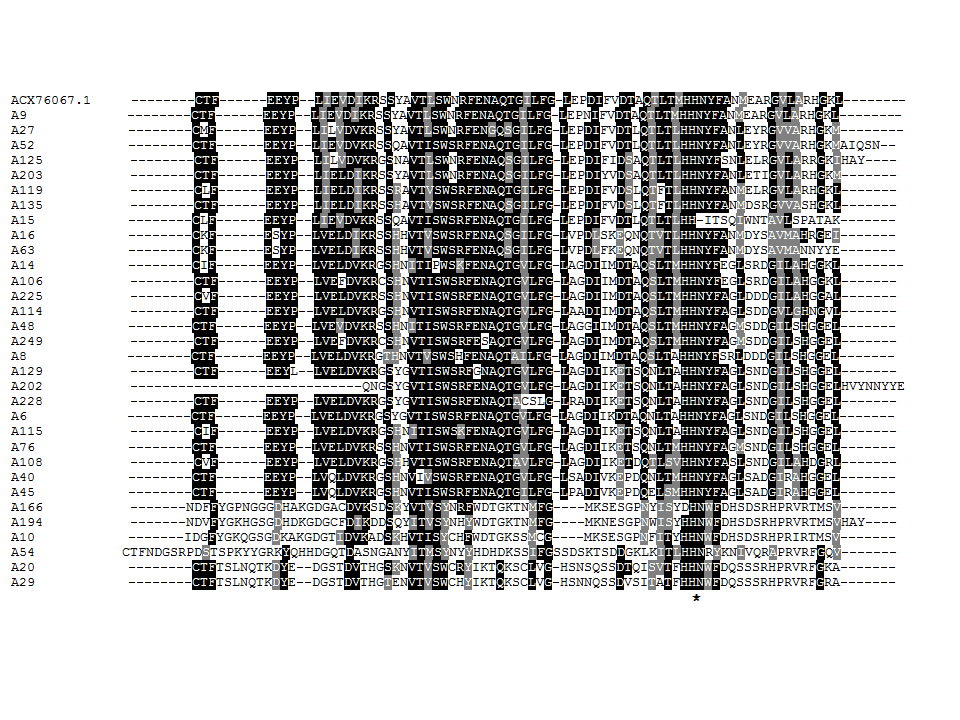

Supplement: Figure S3 — Amino acid sequence alignment by ClustalX of pectinase fragments from clone library of PF00544 with the known pectate lyase from Fibrobacter succinogenes subsp. succinogenes S85 (ACX76067). Identical and similar residues are shaded in black and gray, respectively. The highly conserved residue, His, is indicated with“*”. (TIF) [file pone.0040940.s003.tif]

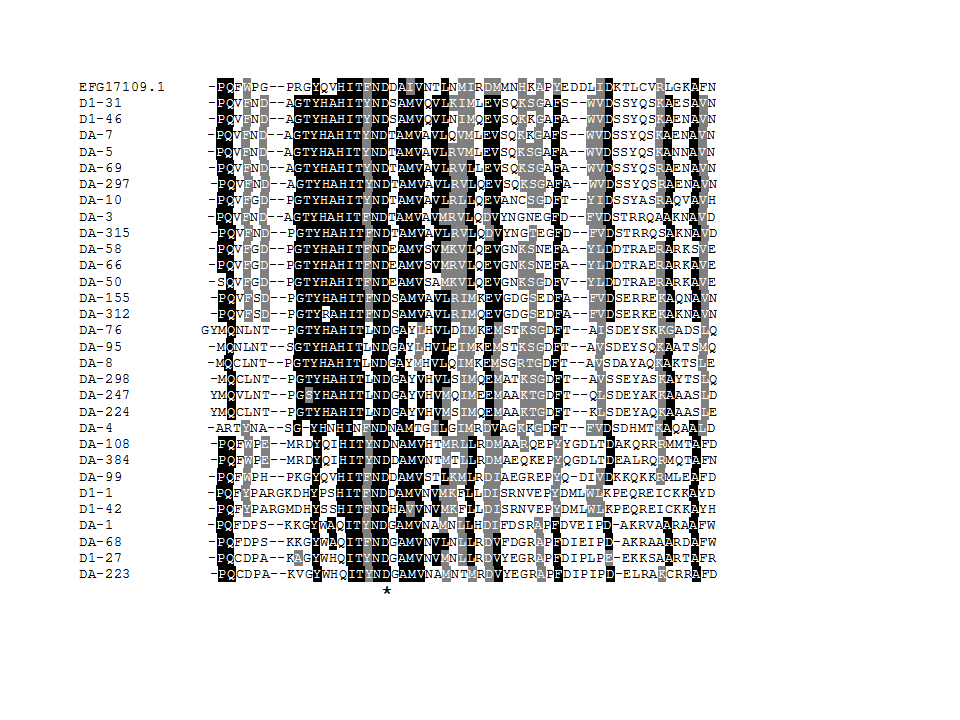

Supplement: Figure S4 — Amino acid sequence alignment by ClustalX of pectinase fragments from clone library of PF09492 with the known pectate lyase from Bacteroides vulgatus PC510 (EFG17109). Identical and similar residues are shaded in black and gray, respectively. The highly conserved residue, Asp, is indicated with “*”. (TIF) [file pone.0040940.s004.tif]
