# Supplementary material for: Abundance and Genetic Diversity of Microbial Polygalacturonase and Pectate Lyase in the Sheep Rumen Ecosystem
Source: PLoS One. 2012 Jul 17;7(7):e40940. doi: 10.1371/journal.pone.0040940 (PMC3398870; doi:10.1371/journal.pone.0040940)
Supplement: Table S1 — PCR-mediated retrieval of known microbial DNA pectinase sequences to test the capacity of the designed primers to retrieve pectinase sequences from the sheep rumen ecosystem. (DOC) [file pone.0040940.s005.doc]

**Table S1.** PCR-mediated retrieval of known microbial DNA pectinase sequences to test the capacity of the designed primers to retrieve pectinase sequences from the sheep rumen ecosystem.

| **Strains^a^** | **Phylum** | **Taxon** | **GenBank and EMBL accession number** | | | | |
| --- | --- | --- | --- | --- | --- | --- | --- |
|  |  |  | **PF00295** | **PF00544** | **PF03211** | **PF06917** | **PF09492** |
| *Alicyclobacillus* sp. CGMCC 3147 | Firmicutes | Gram^+^ | JQ265984 | – | – | – | – |
| *Bacillus* sp. CGMCC 1.2016 | Firmicutes | Gram^+^ | – | JQ265985 | – | – | JQ265986 |
| *Bacillus* sp. B153 | Firmicutes | Gram^+^ | – | – | HE648169 | – | JQ265987 |
| *Streptomyces* sp. ACCC 41168 | Actinobacteria | Gram^+^ | – | JQ265988 | – | – | – |
| *Klebsiella* sp. CGMCC 4433 | Proteobacteria | Gram^–^ | JQ265989 | – | – | JQ265990 | – |
| *Xanthomonas* sp. ACCC 10048 | Proteobacteria | Gram^–^ | JQ265995 | – | – | – | JQ265991 |
| *Erwinia* sp. ACCC 10484 | Proteobacteria | Gram^–^ | – | – | HE648170 | – | – |
| *Erwinia herbicola* 069 | Proteobacteria | Gram^–^ | – | – | HE648171 | – | JQ265992 |
| *Aspergillus* *flavus* J4 | Ascomycota | Fungi | – | – | HE648172 | – | – |
| *Penicillium* sp. CGMCC 1669 | Ascomycota | Fungi | JQ265993 | – | – | – | – |
| *Bispora* sp. CBS 126.38 | Basidiomycota | Fungi | HE648173 | JQ265994 | – | – | – |

^a^All the tested strains are well-separated evolutionarily.
