# Supplementary material for: Abundance and Genetic Diversity of Microbial Polygalacturonase and Pectate Lyase in the Sheep Rumen Ecosystem
Source: PLoS One. 2012 Jul 17;7(7):e40940. doi: 10.1371/journal.pone.0040940 (PMC3398870; doi:10.1371/journal.pone.0040940)
Supplement: Table S2 — Primers used in this study. (DOC) [file pone.0040940.s006.doc]

**Table S2.** Primers used in this study.

| Primer | Sequence (5' → 3')^a^ | Number of bases |
| --- | --- | --- |
| a-cut R | GGCAAGTACTACATGAGCCTGG | 22 |
| A125U1 | GATTTTACCACGACGAGCAAGCACACCGCGAAG | 33 |
| A125U2 | CACACCGCGAAGTTCCAAATTCGAGAAATAATTGTGGTGCAAAGT | 45 |
| A125U3 | CCTCGCTTGACATCAACCAAGATTAGCGGATACTCTTC | 38 |
| A125D1 | TGCACGTTTGAAGAGTATCCGCTAATCTTGGTTGATGTCAAGC | 43 |
| A125D2 | CGCTAATCTTGGTTGATGTCAAGCGAGGCTCGAATG | 36 |
| A125D3 | CGAATTTGGAACTTCGCGGTGTGCTTGCTCGTC | 33 |
| D1U1 | AGCGGATGGCACCGTCGATGGCAAGGTTG | 29 |
| D1U2 | CTCATCGGGATTAGGTAGGGAAAGCAGGAGGTCG | 34 |
| D1U3 | GGATGCATTCCACTCCCCTATCGTATGCCTTCTTGC | 36 |
| D1D1 | TGGACGGACAACCCACGGTGTGGTGTCAG | 29 |
| D1D2 | ACGACGAGCATACGCTCCAGCCTGCTCAAG | 30 |
| D1D3 | ATCGACGGTGCCATCCGCTGGCTCGAG | 27 |
| A125-F | ATGAAAATTAATTGGATGATTGCAGCTCATGTTCGCATGCTTG | 43 |
| A125-R | CTACCGGGGACCGGATTCATTCCTGAC | 27 |
| A125-expF | GCGGTCGACTCGTGATGCCATGGATGACGATAAAGTTGCTGGTG | 44 |
| A125-expR | AAGGAAAAAAGCGGCCGCCTACCGGGGACCGGATTC | 36 |
| D1-F | ATGAGAAAGTCAATAGTTCTTCTCCTTTTC | 30 |
| D1-R | TTATTTTTCGTCATCGTATAAAATATAGTTATCG | 34 |
| D1-expF | TATGATGCCATGGATCAAATACCAGTCGACACGGTCG | 37 |
| D1-expR | CAGGAACAGAAGCGGCCGCTTTTTCGTCATCGTATAAAATATA | 43 |

^a^ Y = C/T, K = T/G, R = A/G, N = A/T/G/C; restriction sites are underlined.
