# Supplementary material for: Abundance and Genetic Diversity of Microbial Polygalacturonase and Pectate Lyase in the Sheep Rumen Ecosystem
Source: PLoS One. 2012 Jul 17;7(7):e40940. doi: 10.1371/journal.pone.0040940 (PMC3398870; doi:10.1371/journal.pone.0040940)
Supplement: Table S3 — Unique PF00295 polygalacturonase gene fragments retrieved from the microbial ecosystem of a Small Tail Han sheep rumen and their closest sequentially related relatives according to amino acid sequence identity. (DOC) [file pone.0040940.s007.doc]

**Table S3. Unique PF00295 polygalacturonase gene fragments retrieved from the microbial ecosystem of a Small Tail Han sheep rumen and their closest sequentially related relatives according to amino acid sequence identity.**

| **OTU^a^** | **Length (aa)** | **Identity (%)** | **Closest relative (GenBank accession number)** | **Sequence abundance (%)** |
| --- | --- | --- | --- | --- |
| a208 | 56 | 52 | *Bacteroides cellulosilyticus* DSM 14838 (ZP_03678536)* | 0.5 |
| a222 | 55 | 55 | *Bacteroides coprocola* DSM 17136 (ZP_03010658) | 0.5 |
| a251 | 56 | 64 | *Bacteroides pectinophilus* ATCC 43243 (ZP_03462639)* | 0.5 |
| a256 | 56 | 64 | *B. pectinophilus* ATCC 43243 (ZP_03462639)* | 0.5 |
| **a38** | **56** | **84** | ***Bacteroides proteoclasticus* B316 (YP_003831820)** | 15.9 |
| a170 | 56 | 71 | *B. proteoclasticus* B316 (YP_003831820) | 1.0 |
| a176 | 56 | 71 | *B. proteoclasticus* B316 (YP_003831820) | 0.5 |
| a199 | 56 | 86 | *B. proteoclasticus* B316 (YP_003831820) | 13.5 |
| a221 | 56 | 70 | *B. proteoclasticus* B316 (YP_003831820) | 5.8 |
| a261 | 47 | 89 | *B. proteoclasticus* B316 (YP_003831820) | 5.3 |
| a269 | 56 | 57 | *B. proteoclasticus* B316 (YP_003831820) | 0.5 |
| a271 | 56 | 68 | *B. proteoclasticus* B316 (YP_003831820) | 14.6 |
| a278 | 56 | 66 | *B. proteoclasticus* B316 (YP_003831820) | 0.5 |
| a63 | 56 | 54 | *Bacteroides* sp. 9_1_42FAA (ZP_04541426) | 2.4 |
| a61 | 56 | 95 | *Butyrivibrio proteoclasticus* B316 (YP_003831820) | 5.3 |
| a91 | 56 | 59 | *Clostridium hathewayi* DSM 13479 (ZP_06114068) * | 0.5 |
| a96 | 56 | 57 | *C. hathewayi* DSM 13479 (ZP_06114068)* | 1.4 |
| a187 | 56 | 68 | *C. hathewayi* DSM 13479 (ZP_06114068) * | 0.5 |
| a267 | 57 | 79 | *C. hathewayi* DSM 13479 (ZP_06114068) * | 2.5 |
| a236 | 56 | 54 | *C. hathewayi* DSM 13479 (ZP_06114068)* | 0.5 |
| a89 | 56 | 70 | *Clostridium asparagiforme* DSM 15981 (ZP_03762690) | 0.5 |
| a220 | 56 | 54 | *Clostridium phytofermentans* ISDg (YP_001559836) | 2.4 |
| a286 | 56 | 59 | *C. phytofermentans* ISDg (YP_001559836) | 2.4 |
| a284 | 56 | 55 | *C. phytofermentans* ISDg (YP_001559836) | 1.0 |
| a62 | 56 | 64 | *Ruminococcus albus* 8 (ZP_08159485) | 0.5 |
| a180 | 56 | 63 | *R. albus* 8 (ZP_08159485) | 1.5 |
| a55 | 55 | 58 | *Roseburia intestinalis* XB6B4 (CBL11994.1) | 1.9 |
| a69 | 56 | 55 | *R. intestinalis* XB6B4 (CBL11994.1) | 0.5 |
| a156 | 56 | 82 | *R. intestinalis* XB6B4 (CBL11994) | 0.5 |
| a173 | 56 | 73 | *R. intestinalis* XB6B4 (CBL11994) | 0.5 |
| a8 | 54 | 52 | *Thermoanaerobacter italicus* Ab9 (YP_003477542) | 0.5 |
| a234 | 56 | 54 | *Thermotoga thermarum* DSM 5069 (YP_004659588) | 1.5 |
| a147 | 56 | 84 | *B. proteoclasticus* B316 (YP_003831820) | 2.9 |
| a143 | 56 | 64 | *C. hathewayi* DSM 13479 (ZP_06114068)* | 0.5 |
| a142 | 56 | 80 | *C. hathewayi* DSM 13479 (ZP_06114068)* | 0.5 |
| a135 | 56 | 84 | *B. proteoclasticus* B316 (YP_003831820) | 2.9 |
| a125 | 56 | 82 | *B. proteoclasticus* B316 (YP_003831820) | 2.4 |
| a124 | 56 | 63 | *C. phytofermentans* ISDg (YP_001559836) | 0.5 |
| a115 | 56 | 66 | *Roseburia intestinalis L1-82* (ZP_04742697)* | 1.9 |
| a101 | 56 | 55 | *R. intestinalis* XB6B4 (CBL11994.1) | 1.0 |
| a107 | 56 | 68 | *C. hathewayi* DSM 13479 (ZP_06114068)* | 1.0 |

^a^Operational taxonomic unit. The most abundant gene fragment of 213 clones sequenced is in bold type.

*Hypothetical protein.
