# Supplementary material for: Abundance and Genetic Diversity of Microbial Polygalacturonase and Pectate Lyase in the Sheep Rumen Ecosystem
Source: PLoS One. 2012 Jul 17;7(7):e40940. doi: 10.1371/journal.pone.0040940 (PMC3398870; doi:10.1371/journal.pone.0040940)
Supplement: Table S4 — Unique PF00544 pectate lyase gene fragments retrieved from the microbial ecosystem of a Small Tail Han sheep rumen and their closest sequentially related relatives according to amino acid sequence identity. (DOC) [file pone.0040940.s008.doc]

**Table S4. Unique PF00544 pectate lyase gene fragments retrieved from the microbial ecosystem of a Small Tail Han sheep rumen and their closest sequentially related relatives according to amino acid sequence identity.**

| **OTU^a^** | **Length (aa)** | **Identity (%)** | **Closest relative (GenBank accession number)** | **Sequence abundance (%)** |
| --- | --- | --- | --- | --- |
| A54 | 90 | 100 | *Bacillus subtilis* subsp. *natto* BEST195 (BAI84271) | 0.5 |
| A6 | 72 | 67 | *Fibrobacter succinogenes* subsp. *succinogenes* S85 (YP_003250549) | 12.4 |
| A8 | 72 | 61 | *F. succinogenes* subsp. *succinogenes* S85 (YP_003250549) | 1.1 |
| A9 | 72 | 99 | *F. succinogenes* subsp. *succinogenes* S85 (YP_003250549) | 1 |
| A14 | 72 | 63 | *F. succinogenes* subsp. *succinogenes* S85 (YP_003250549) | 5.9 |
| A15 | 71 | 66 | *F. succinogenes* subsp. *succinogenes* S85 (YP_003250549) | 2.1 |
| A16 | 72 | 63 | *F. succinogenes* subsp. *succinogenes* S85 (YP_003250549) | 3.2 |
| A27 | 72 | 85 | *F. succinogenes* subsp. *succinogenes* S85 (YP_003250549) | 3.2 |
| A40 | 72 | 61 | *F. succinogenes* subsp. *succinogenes* S85 (YP_003250549) | 0.5 |
| A45 | 72 | 63 | *F. succinogenes* subsp. *succinogenes* S85 (YP_003250549) | 5.9 |
| A48 | 72 | 68 | *F. succinogenes* subsp. *succinogenes* S85 (YP_003250549) | 5.9 |
| **A52** | **77** | **82** | ***F. succinogenes* subsp. *succinogenes* S85 (YP_003250549)** | **14.1** |
| A63 | 72 | 63 | *F. succinogenes* subsp*. succinogenes* S85 (YP_003250549) | 3.2 |
| A76 | 72 | 67 | *F. succinogenes* subsp*. succinogenes* S85 (YP_003250549) | 0.5 |
| A106 | 72 | 68 | *F. succinogenes* subsp*. succinogenes* S85 (YP_003250549) | 5.9 |
| A108 | 72 | 61 | *F. succinogenes* subsp. *succinogenes* S85 (YP_003250549) | 0.5 |
| A114 | 72 | 68 | *F. succinogenes* subsp*. succinogenes* S85 (YP_003250549) | 2.1 |
| A115 | 72 | 58 | *F. succinogenes* subsp. *succinogenes* S85 (YP_003250549) | 0.5 |
| A119 | 72 | 86 | *F. succinogenes* subsp. *succinogenes* S85 (YP_003250549) | 0.5 |
| **A125** | **75** | 83 | ***F. succinogenes* subsp*. succinogenes* S85 (YP_003250549)** | **13.5** |
| A129 | 72 | 61 | *F. succinogenes* subsp*. succinogenes* S85 (YP_003250549) | 0.5 |
| A135 | 72 | 82 | *F. succinogenes* subsp. *succinogenes* S85 (YP_003250549) | 1.6 |
| A202 | 67 | 57 | *F. succinogenes* subsp. *succinogenes* S85 (YP_003250549) | 5 |
| A203 | 72 | 88 | *F. succinogenes* subsp. *succinogenes* S85 (YP_003250549) | 0.5 |
| A225 | 72 | 68 | *F. succinogenes* subsp. *succinogenes* S85 (YP_003250549) | 1 |
| A228 | 72 | 60 | *F. succinogenes* subsp. *succinogenes* S85 (YP_003250549) | 0.5 |
| A249 | 72 | 67 | *F. succinogenes* subsp. *succinogenes* S85 (YP_003250549) | 0.5 |
| A166 | 78 | 78 | *Prevotella bergensis* DSM 17361 (ZP_06005210) | 1.6 |
| A194 | 81 | 73 | *P. bergensis* DSM 17361 (ZP_06005210) | 1.6 |
| A10 | 78 | 85 | *Prevotella bryantii* B14 (ZP_07061110) | 3.2 |
| A20 | 78 | 49 | *Streptomyces violaceusniger* Tu 4113 (ZP_07603961) | 0.5 |
| A29 | 78 | 42 | *S. violaceusniger* Tu 4113 (ZP_07603961) | 0.5 |

^a^Operational taxonomic unit. The most abundant gene fragment of 187 clones sequenced and the gene fragment used to clone *A125* are in bold type.

*Hypothetical protein.
