# Supplementary material for: Abundance and Genetic Diversity of Microbial Polygalacturonase and Pectate Lyase in the Sheep Rumen Ecosystem
Source: PLoS One. 2012 Jul 17;7(7):e40940. doi: 10.1371/journal.pone.0040940 (PMC3398870; doi:10.1371/journal.pone.0040940)
Supplement: Table S5 — Unique PF09492 pectate lyase gene fragments retrieved from the microbial ecosystem in a Small Tail Han sheep rumen and their closest sequentially related relatives according to amino acid sequence identity. (DOC) [file pone.0040940.s009.doc]

**Table S5. Unique PF09492 pectate lyase gene fragments retrieved from the microbial ecosystem in a Small Tail Han sheep rumen and their closest sequentially related relatives according to amino acid sequence identity.**

| **OTU^a^** | **Length (aa)** | **Identity (%)** | **Closest relative (GenBank accession number)** | **Sequence abundance (%)** |
| --- | --- | --- | --- | --- |
| DA-108 | 75 | 65 | *Bacteroides cellulosilyticus* DSM 14838 (ZP_03679297) | 0.7 |
| DA-384 | 75 | 69 | *B. cellulosilyticus* DSM 14838 (ZP_03679297) | 0.7 |
| DA-1 | 74 | 62 | *Bacteroides clarus* YIT 12056 (ZP_08297969) | 2.1 |
| DA-68 | 74 | 61 | *Bacteroides coprocola* DSM 17136 (ZP_03009633) | 2.1 |
| D1-27 | 74 | 61 | *Bacteroides eggerthii* 1_2_48FAA (ZP_07934415) | 0.7 |
| DA-223 | 74 | 55 | *B. eggerthii* 1_2_48FAA (ZP_07934415) | 0.7 |
| DA-4 | 72 | 51 | *Clostridium cellulovorans* 743B (YP_003845268) | 1.4 |
| DA-247 | 74 | 47 | *C. cellulolyticum* H10 (YP_002505581) | 1.4 |
| DA-8 | 69 | 43 | *Clostridium papyrosolvens* DSM 2782 (ZP_08191337) | 2.1 |
| DA-50 | 73 | 59 | *C. papyrosolvens* DSM 2782 (ZP_08191337) | 0.7 |
| DA-58 | 73 | 59 | *C. papyrosolvens* DSM 2782 (ZP_08191337) | 1.4 |
| DA-66 | 73 | 59 | *C. papyrosolvens* DSM 2782 (ZP_08191337) | 2.7 |
| DA-76 | 81 | 49 | *C. papyrosolvens* DSM 2782 (ZP_08191337) | 1.4 |
| DA-95 | 73 | 51 | *C. papyrosolvens* DSM 2782 (ZP_08191337) | 0.7 |
| DA-224 | 74 | 47 | *C. papyrosolvens* DSM 2782 (ZP_08191337) | 0.7 |
| DA-298 | 73 | 53 | *C. papyrosolvens* DSM 2782 (ZP_08191337) | 0.7 |
| **D1-1** | **77** | 44 | ***Cyclobacterium marinum* DSM 745 (YP_004774859)** | **40** |
| D1-42 | 77 | 43 | *C. marinum* DSM 745 (YP_004774859) | 0.7 |
| DA-99 | 75 | 60 | *Prevotella bergensis* DSM 17361 (ZP_06005091)* | 0.7 |
| DA-3 | 73 | 60 | *Ruminococcus albus* 7 (YP_004104239) | 8.2 |
| DA-5 | 73 | 88 | *R. albus* 7 (YP_004104239) | 17 |
| DA-7 | 73 | 89 | *R. albus* 7 (YP_004104239) | 2.1 |
| DA-10 | 73 | 66 | *R. albus* 7 (YP_004104239) | 0.7 |
| D1-31 | 73 | 95 | *R. albus* 7 (YP_004104239) | 0.7 |
| D1-46 | 73 | 90 | *R. albus* 7 (YP_004104239) | 2.1 |
| DA-69 | 73 | 86 | *R. albus* 7 (YP_004104239) | 2.7 |
| DA-155 | 73 | 70 | *R. albus* 7 (YP_004104239) | 0.7 |
| DA-297 | 73 | 88 | *R. albus* 7 (YP_004104239) | 2.7 |
| DA-312 | 73 | 67 | *R. albus* 7 (YP_004104239) | 0.7 |
| DA-315 | 73 | 60 | *R. albus* 7 (YP_004104239) | 0.7 |

^a^Operational taxonomic unit. The most abundant gene fragment of 172 clones sequenced is shown in bold type. This fragment was used to clone *D1-1*.

*Hypothetical protein.
