# Supplementary material for: Abundance and Genetic Diversity of Microbial Polygalacturonase and Pectate Lyase in the Sheep Rumen Ecosystem
Source: PLoS One. 2012 Jul 17;7(7):e40940. doi: 10.1371/journal.pone.0040940 (PMC3398870; doi:10.1371/journal.pone.0040940)
Supplement: Table S6 — Percentages of total PF00295, PF00544, and PF09492 fragments (operational taxonomic units) that have amino acid sequence identities within a given range in comparison with GenBank pectinase sequences. (DOC) [file pone.0040940.s010.doc]

**Table S6.** Percentages of total PF00295, PF00544, and PF09492 fragments (operational taxonomic units) that have amino acid sequence identities within a given range in comparison with GenBank pectinase sequences.

| **Pectinase family** | **Range of amino acid identities (%)** | | | |
| --- | --- | --- | --- | --- |
|  | **>81%** | **66–80%** | **51–65%** | **<50%** |
| PF00295 | 17.1 | 26.8 | 51.2 | 4.9 |
| PF00544 | 33.3 | 24.2 | 39.5 | 3.0 |
| PF09492 | 20.0 | 10.0 | 53.3 | 16.7 |
